# Counselling toward reducing alcohol use, knowledge about its morbidity and personal consumption among students of medical and dental courses in north-western Spain

**DOI:** 10.4317/medoral.24950

**Published:** 2021-10-27

**Authors:** Alba Pérez-González, Alejandro I Lorenzo-Pouso, Pilar Gándara-Vila, Andrés Blanco-Carrión, José M Somoza-Martín, Tamara García-Carnicero, Mario Pérez-Sayáns

**Affiliations:** 1 Oral Medicine, Oral Surgery and Implantology, Unit (MedOralRes). Faculty of Medicine and Dentistry, Universidade de Santiago de Compostela, Spain; 2 ORALRES group. Instituto de Investigación Sanitaria de Santiago (IDIS), Santiago de Compostela, Spain

## Abstract

**Background:**

Alcohol use disorder (AUD) is directly linked to high-risk consumption. Healthcare students have a crucial role to play in its prevention and management. The aim of this study is to analyse alcohol consumption, as well as to consider the knowledge and attitudes regarding morbidity, and the stage of change when providing assistance to quit AUD.

**Material and Methods:**

A cross-sectional study was conducted among Dentistry and Medical students using specific and validated questionnaires in an anonymous and voluntary way. Initially, 925 students were invited to participate, of them 500 were reached.

**Results:**

Among them 85.9% suffered from AUD of whom 75% were women (*p*<0.001), and it was considered that the female gender constituted an independent risk factor (OR=2.63, CI 95% 1.55-4.45, *p*<0.001). The majority of the participants did not achieve the pass mark, nonetheless, the results showed improved levels of knowledge among participants in the latter years of their studies (*p*<0.001). Dental students demonstrated greater shortcomings in terms of their knowledge of general pathology, whereas the medical students’ knowledge of oral pathologies proved worse (*p*<0.001). Most of students believed that identifying cases of AUD-affected patients falls within their competence, nonetheless, they believed that they do not have the necessary competencies. Among participants 58.2% were in a stage of change regarding AUD attitudes.

**Conclusions:**

The majority of respondents presented AUD. In general, the participants’ knowledge about alcohol was low. Reviewing the syllabuses and evaluating the implementation of gender-differentiated training programmes in both degrees would be considered necessary.

** Key words:**Alcohol use disorder, healthcare students, addiction, health literacy, behaviours.

## Introduction

Alcohol use disorder (AUD) refers to a long-term alcohol addiction that is linked to mental and/ or physical problems, and which is characterised by compulsive consumption, loss of control over consumption, and a negative emotional state when not using alcohol (1). According to the "Global status report on alcohol and health", which was published by the WHO in 2018, the harmful use of alcohol resulted in around 3 million deaths (5.3 per cent of all deaths) worldwide that year as well as 132.6 million "disability-adjusted life years" (DALY) -the number of years lost due to illness, disability or premature death-, that is to say, 5.1 per cent of all of the DALYs in that year. The European Union has the highest rate of alcohol consumption in the world (10.2 litres of pure alcohol per person per year).

Alcohol has been classified by the International Agency for Research on Cancer (IARC) as a Group 1 carcinogen; and in particular, a strong relationship has been detected between alcohol consumption and head and neck cancer (HNC). Each unit of alcohol consumed increases the incidence of HNC by 1 per 1000 (2) . Different *in vivo* and *in vitro* studies have presented the influence that alcohol consumption has on cancer of the oral cavity and on the transition from potentially malignant oral disorders to oral cancer (3). From the perspective of public health, several groups of health professionals have developed interventions which aim to reduce alcohol consumption and likewise this problem has also been addressed in some HNC detection projects (4).

Oral health professionals can play a crucial role in the early diagnosis of oral cancer, which represents a particularly relevant group (5). These professionals can provide brief interventions at the primary care level. However, there is no literature which seeks to optimise or collect specific information on AUD or alcohol prevention strategies in this subgroup, whereas in recent times, emphasis has been placed on other modifiable risk factors relevant to oral carcinogenesis (such as HPV infection) (6).

The study of alcohol consumption among university students has generated considerable interest throughout the world, and the first research on this topic in industrialised countries was performed in the 1970s and showed the high levels of consumption and the problems associated with this excessive use since the 1980s (7). Subsequently, several epidemiological studies have revealed a high prevalence of alcohol consumption among university students (8). Very limited research has provided information on the prevalence and intensity of alcohol consumption specifically among dentistry and medical students.

Our working hypothesis considered that consumption levels in the faculties of medicine and dentistry are similar to those observed in the general population for the same age group. Therefore, the aims of this study were: 1) to analyse the alcohol consumption of dentistry and medical students at the University of Santiago de Compostela (USC) as well as their knowledge of the effects of said consumption and 2) to study the stage of change which they are in with regards to helping patients to give up alcohol.

## Material and Methods

- Design of the study

This cross-sectional study was conducted in the Faculties of Medicine and Dentistry of the University of Santiago de Compostela (Spain) following the STROBE guidelines for observational studies (9). The bioethics committee of the USC gave consent for its execution (Ref. AUC-19-2019). All of the first to the fifth year and postgraduate dentistry students, and the first and final year medical students were invited to participate. Printed surveys were distributed during teaching hours and participation was anonymous and voluntary. The survey was also delivered by means of a Google form which was sent to students who were unable to attend the on-site classes.

- Validation of the instrument

A pilot survey was tested on a sample of 50 randomly selected students in order to assess the reliability and thoroughness of the survey, as well as the time taken to complete it. The average time taken to complete the survey was seven minutes. These 50 pilot cases were subsequently discarded and the final sample was collected. Cronbach's alpha test was used to determine the final reliability of the test. Stratification in thematic blocks was used and the weighted mean value for this survey was 0.74. The data allowed us to ensure moderate reliability.

- Questionnaire

The survey was divided into six sections: (i) Sociodemographic data (age, gender, year of study and faculty), (ii) Individual alcohol consumption, (iii) Ease with which they deal with sensitive issues during clinical practice, (iv) Knowledge about alcohol morbidity, (v) Knowledge of alcohol prevention strategies and (vi) Stages of change. The second section used the validated version of the AUDIT-C (increasing the cut-off point by one unit, which therefore increased its predictive value, bringing it to 4 in men and 5 in women) (10) and the CAGE test, validated by Mayfield in 1974 (11). In sections III and V, the 5-point Likert scale was used with the questions on attitude and confidence described by Ntouva *et al*. (12). Section IV combined two multiple choice questions and eight single-answer questions about alcohol knowledge as described by Miller *et al*. (13). The last section (VI) measured the stages of change. This section was based on the transtheoretical model which was designed by Prochaska & DiClemente, and which the authors had adapted in 2019 based on a previous adaptation by Lorenzo-Pouso *et al*. (6). Although this version looked to analyse professional health attitudes in relation to providing advice to patients regarding the HPV infection, whereas the adapted version used for this study looked to evaluate the readiness of medical and dental students to provide advice to patients on giving up alcohol. This last section enabled us to divide students into four groups: pre-contemplation, contemplation, action and change.

- Sample size

In order to calculate the minimum sample size required, we used Epidat 4.2 (SERGAS, Galicia, Spain) with a confidence interval of 95% and a margin of error of 5%. The sample size calculation was based on the number of enrolled students at that time (n=225 in Dentistry) and (n=700 in Medicine). Based on this number, the minimum sample size required for our design was 275, and in the end we included 500 surveys.

- Variables and collected data

Demographic data: age, gender, faculty, year of study, cycle (first and second year students corresponded to the first cycle; third, fourth and fifth year to the second cycle and the postgraduate degrees in dentistry and the sixth year of the medical degree corresponded to postgraduate studies). Alcohol consumption: AUDIT- C, Sum AUDIT-C, Risk Drinking (High/Low), CAGE (C: Cut down; A: Annoyed; G: Guilty; E: Eye opener), Sum CAGE. Attitudes (Likert Scale). Knowledge about alcohol (test) and its morbidity in oral and general pathologies (multiple choice). Trust levels (Likert scale). Stages of change. Weighted (0-10) and grouped grades (fail, pass, good, outstanding).

- Statistical analysis

The data was collected in a specifically designed database and it was manually entered with repeated control in order to avoid errors. This data was statistically analysed using the SPSS v.24.0 software (IBM, Statistics, NY, USA). The categorical variables were described by frequencies and percentages, and the quantitative variables were described by mean and standard deviation. According to the central limit theorem, we considered the distribution of quantitative variables to be normal given the large sample size. Contingency Tables were established in order to study the relationships between the categorical variables using the Chi-square test. Parametric statistics were used to study the effect of the quantitative variables on the qualitative variables, performing the ANOVA test with Bonferroni post-hoc correction for comparisons with more than two elements. We analysed the paired correlation of quantitative variables using Pearson's coefficient. Binomial logistic regression analysis was performed in order to verify the risk of AUD. The significance level was set at p≤0.05.

## Results

- Descriptive

The sample consisted of 500 participants with a mean age of 20.7±3.8 years. 134 of the participants were men (26.8%), 361 were women (72.2%) and 5 were self-included in the gender: other (1%). The response rate was 47% in the faculty of medicine and 74.2% in the faculty of dentistry. 420 surveys were handed out on site, of which 394 were returned, and 500 were sent online, with 106 responses. Participation in the virtual survey was lower and mostly female (74.3%). Full descriptive data can be found in Table 1.

- Alcohol consumption

The AUDIT-C showed an average value of (5.72±2.06) with a range of 3-15. By turning this variable into low and high-risk drinkers, we observed that 75.5% of the at-risk drinkers from our sample were women, compared to 24.1% of men, which was considered to be a statistically significant difference (*p*<0.001) (Table 2). However, when using the CAGE test no statistically significant differences regarding consumption were observed. By performing a binomial logistic regression, we verified the role of gender as the only independent factor for suffering from AUD, and in particular women have an accentuated risk (OR=2.63, CI:95% 1.55-4.45, *p*<0.001).

- Knowledge

Overall the participants’ knowledge on alcohol morbidity was considered to be low. In terms of general pathology, the knowledge of more than half of the students from both faculties was not considered to be satisfactory. 55.7% of the students from the faculty of dentistry and 51.7% of students from the faculty of medicine failed, and just 1.2% and 5.1% of the students from the respective faculties were awarded the score of outstanding (*p*=0.020). With regards to the cycle, almost 60% of undergraduate students failed compared with 36.6% of the postgraduate students (*p*<0.001). In terms of oral pathology, 67.9% of the male students failed in comparison with 64.3% of the female students (*p*=0.044). In this specific field, 73% of the students from the faculty of medicine failed, in comparison with 49.1% of the students from the faculty of dentistry (*p*<0.001), and more than 80% of students from the first cycle failed compared to 29.5% of students from the second cycle (*p*<0.001).

With regards to the quantitative analysis of the students’ weighted knowledge, the results were similar to the grouped results and we found significant differences in the students’ knowledge of general and oral pathology depending on which academic cycle they were in. With regards to their knowledge about general pathology, the average score for students in the second cycle of 5.58 (CI: 95% 5.24-6.10) was higher than that of the students in the first cycle in which the average of 4.52 represented a fail grade (CI: 95% 4.31-4.72) (*p*<0.001). With regards to knowledge of oral pathology, the average mark for the dentistry students was 5.09, which represented a pass grade (CI: 95% 4.76-5.42) while the average mark for medical students was 4.02, representing a fail grade (CI: 95% 3.75-4.27) (*p*<0.001). We were able to appreciate a greater difference in the students’ knowledge of oral pathology when taking their level of study into account, than we did when we made the same comparison when considering their knowledge of general pathology. In the latter the second cycle students achieved higher marks (6.03, CI:95% 5.57-6.49) than the students in the first cycle (3.60, CI:95% 3.40-3.80) (*p*<0.001). However, these differences were not appreciated in terms of their knowledge of alcohol, which in general was low, and was only minimally accepTable amongst the students from the second cycle and without any statistically significant differences.

**Table 1:**
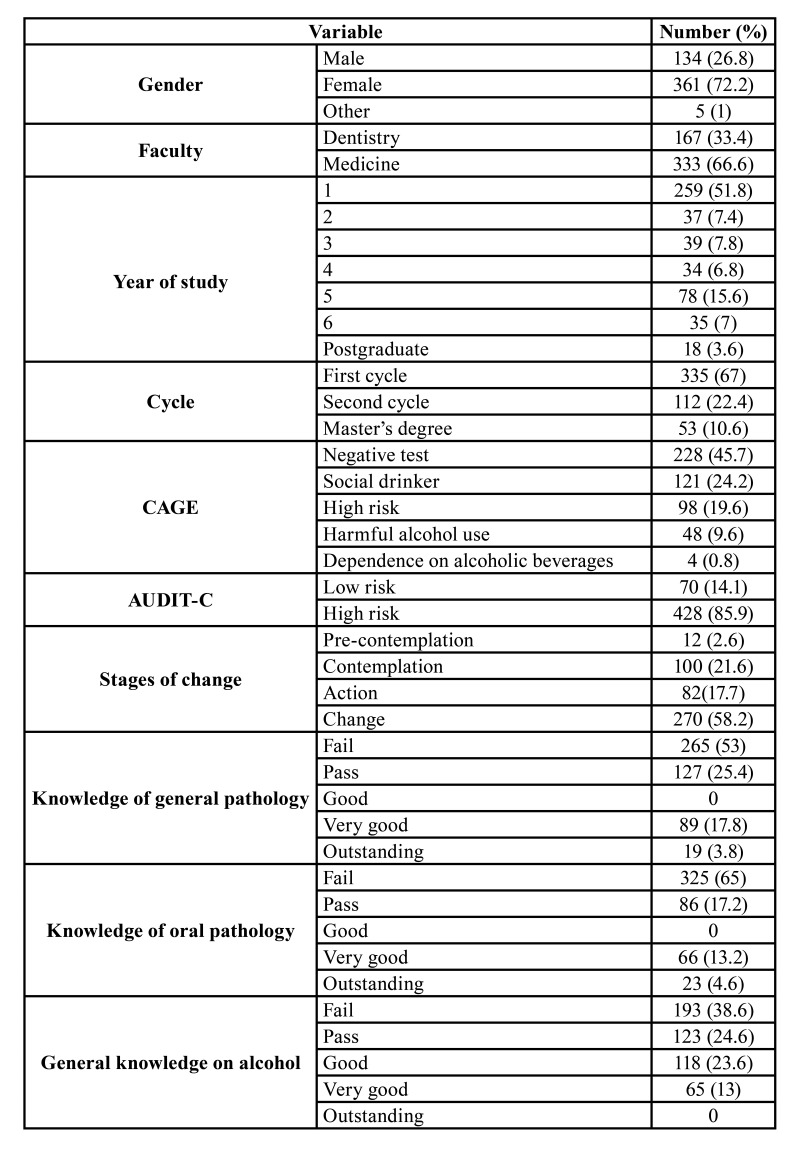
Descriptive data of the sample.

**Figure 1:**
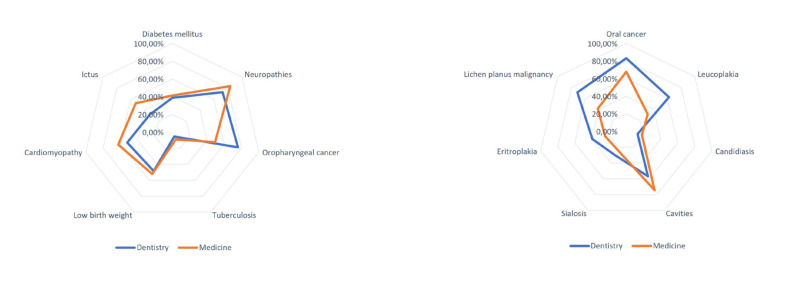
Spider web diagram on knowledge of alcohol morbidity: A) General pathology, B) Oral pathology.

In Fig. 1 is displayed the data related to the specific matters for each field. The main pathologies that the students associated with alcohol consumption were: neuropathies (n=399), followed by oral cancer (n=365) and cavities (n=343). The least associated were: tuberculosis (n=39) and candidiasis (n=89). With regards to their general knowledge about alcohol, no statistically significant differences were observed, with 61.3% of the individuals presenting sufficient knowledge on this topic.

- Stages of change

Broadly speaking, most of the participants were in a stage of change (58.2%) (Table 3). This was the case for 63.3% of the medical students and 49.1 % of the dentistry students (*p*<0.001). However, with regards to the cycle, 62% of the students in the first cycle were in a stage of change, compared with 56% of students in the second cycle and 40.4% of the master’s degree students (*p*=0.024) respectively.

Table 4 shows the relationship between the scores obtained in the different knowledge areas and the influence of these on the stages of change. Here we can observe that as the students’ knowledge of general pathology improves, so too does their stage. As such, students in the pre-contemplation stage achieved an average mark of 3.10, (CI:95% 2.33-3.85) and this increased amongst students in the action (5.05, CI:95% 4.59-5.51, *p*=0.015) and change stages (5.08, CI:95% 4.81-5.34, *p*=0.008) with an average pass mark. Statistically significant differences were not observed in the rest of the knowledge areas.

**Table 2:**
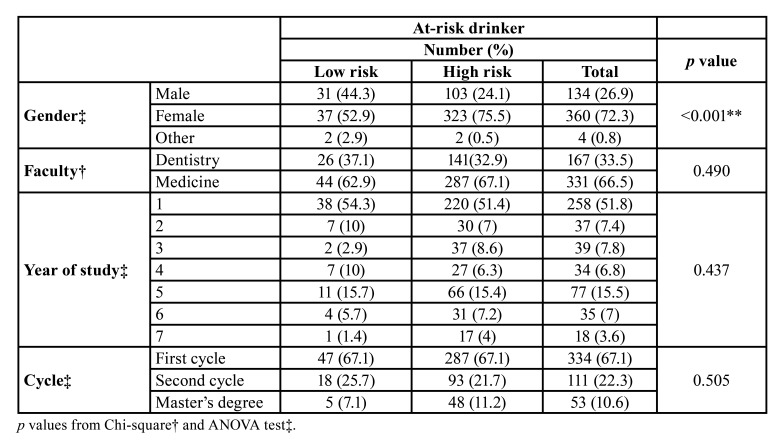
Analysis of risk of consumption according to gender, faculty, year of study and cycle.

**Table 3:**
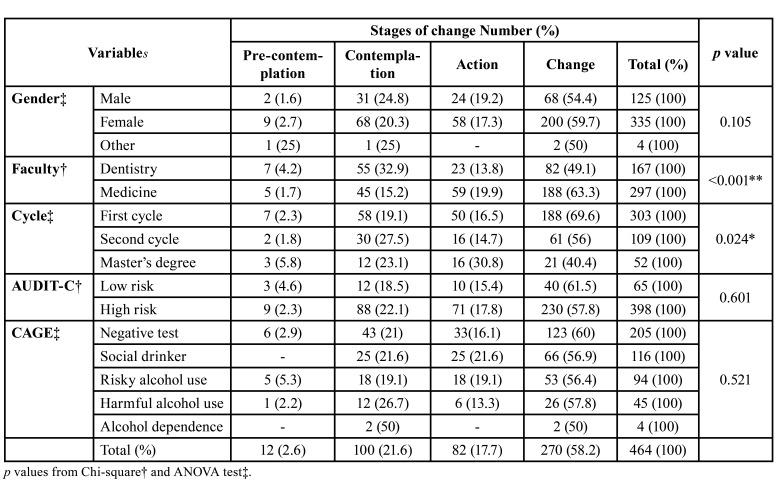
Distribution of the stages of change according to Prochaska-DiClement model.

**Table 4:**
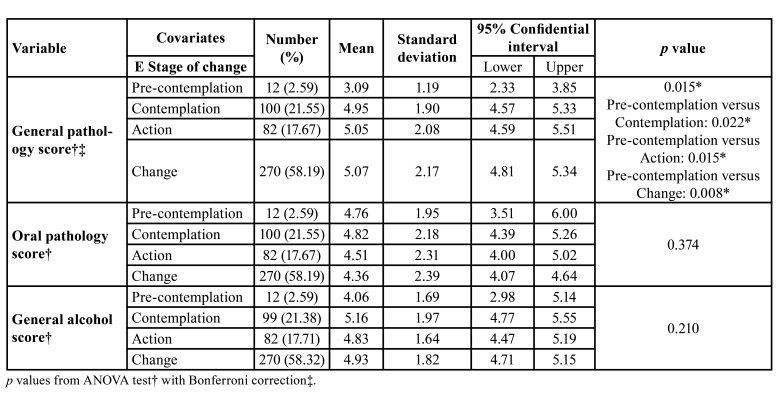
Analysis of knowledge of alcohol morbidity according to the stage of change.

- Attitudes

**Figure 2:**
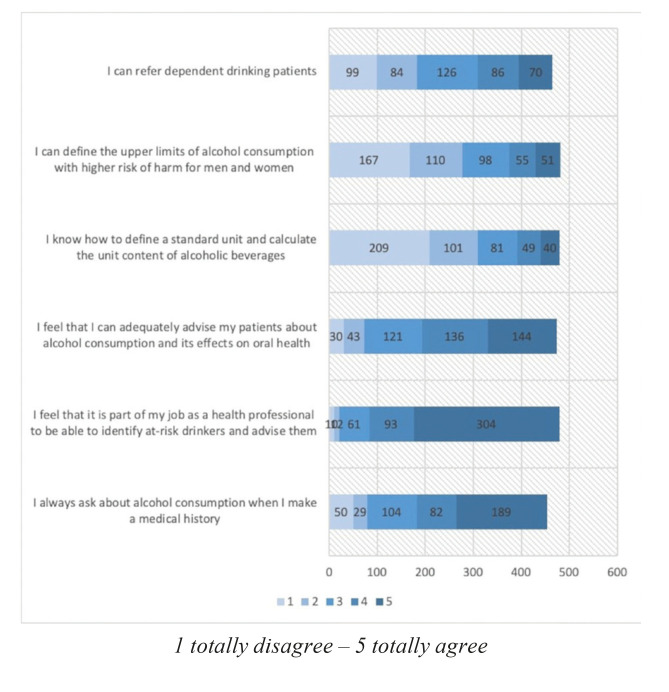
Attitudes and perceptions.

With regards to the students’ attitudes, the majority stated that they ask their patients about alcohol consumption when taking their clinical history, and 60.8% completely agreed that identifying AUD and assisting them with this matter forms part of their job. A high percentage of the participants felt that they were able to do so in an adequate manner, however, a high number of the participants were neither able to determine the upper limits of consumption for men and women, nor were they able to calculate the consumption in standard units (only 8% claimed that they definitely knew how to define a standard unit) as we can see in Fig. 2.

**Figure 3:**
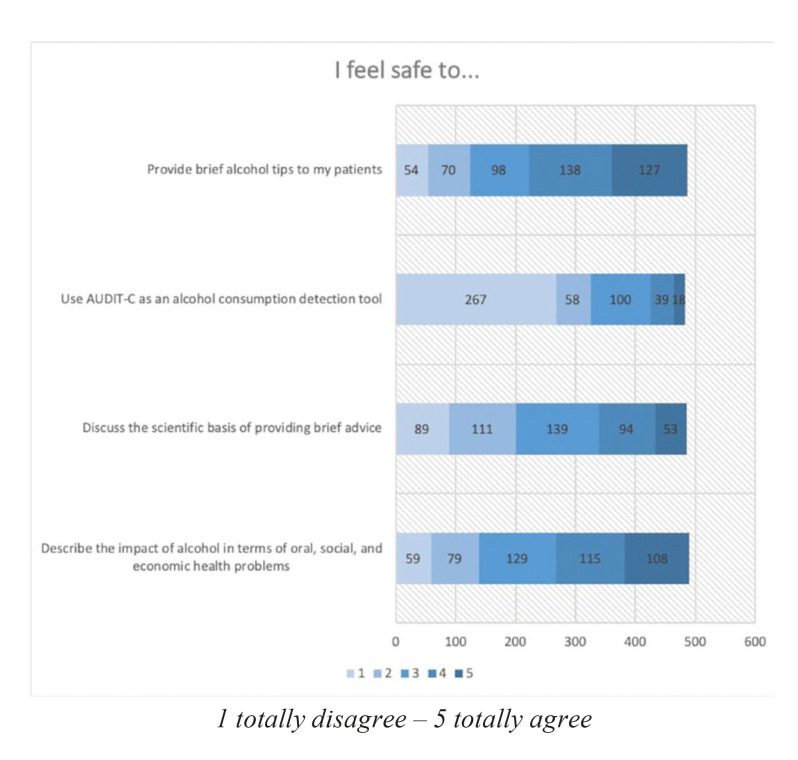
Level of confidence.

With regard to the participants’ levels of confidence (Fig. 3), although a high percentage of the students considered that they were capable of giving advice, very few felt that they were able to discuss the scientific basis of the advice or the effects of alcohol. 41.2% stated that they did not feel at all confident to do so, or that they did not know how to use the AUDIT-C test to detect cases of risk.

## Discussion

Alcohol consumption within our sample proved alarming, as according to the AUDIT-C test, 85.9% of the participants were at-risk drinkers and this high prevalence has also been reported among health science students in Spain by Rabanales Sotos *et al*. (14). Our results are perfectly comparable with the data gathered in Europe: Ireland (65.2% of men and 67.3% of women presented AUD) (15) and UK (86.1% are consumers and more than a half presented AUD) (16); even globally: Tunisia (52.5% of students presented AUD) (17), Ecuador (49.73% of men and 23.80% of women reported AUD) (18), and Australia (almost 25% present risky alcohol use, higher in the case of men) (19). It is worth highlighting that 75.5% of the at-risk drinkers in our study were women. However, when comparing the results in depth, we observed that the data regarding the prevalence of “at-risk” consumption is heterogeneous due to the variability of the definitions used by the authors, the measurement tools and the established thresholds. In our opinion, global standard protocols and guidelines must be established in order to determine the risk of AUD.

On the other hand, we also studied the knowledge of the medical and dentistry students regarding alcohol-related pathologies given that these are fundamental strategies in preventive medicine. The participants’ knowledge of oral and general pathologies was considered to be insufficient in both levels, and likewise, significant differences were observed between the cycles. This divergence can be explained by the acquisition of specific knowledge throughout the degree programme. Nevertheless, even in the latter years of study, the scores in general were low. Our results were similar to those of the study published by Wang *et al*. in 2020 (20) in which the majority of health students in Mongolia demonstrated very limited knowledge about the damage caused by alcohol, and this lack of knowledge was greater in occasional and regular drinkers than it was in non-drinkers (74.0% and 75.3% vs 42.1%). Kujan *et al*. studied the knowledge about oral cancer among students in Arabia Saudi and conveyed the need for the undergraduate study syllabus to be reinforced, in particular with regards to education on oral cancer, and specifically its prevention and early detection (21,22). 

Therefore, it is clear that initiatives must be introduced in order to reduce alcohol abuse and the damage associated with it. These initiatives must focus on changing social beliefs and standards, as well as looking into the factors that affect the decisions made by young people to consume alcohol (16). These programs must be implemented according to gender (18), focus on the different stages which the students find themselves in, and likewise, they must be implemented in conjunction with effective public policies that can address the problem.

In recent years, different groups have worked on dental education projects related to the oral pathology, directed both at professionals from the sector and dentistry students (6,21,22). In the USA, a three-week pilot program in which medical students treated patients suffering from mental disorders caused by substance use led to students feeling more confident and at ease when treating these patients and these results were sustained over time (23). In particular, and in relation to alcohol, Farah *et al*. published some of the most relevant references showing the relationship between this addictive behaviour and oral cancer (24).

With regards to attitudes and perceptions, a considerable lack of documentation can be observed. In our sample, the majority of participants were aware that identifying and helping at-risk drinkers is fundamental, however, the results showed that most of them did not feel that they were prepared to do so. In a study conducted among medical students in Lebanon, 38.2% of the students felt that they had received the appropriate training on AUD and just 29.1% showed any interest in working in this field. Around 75% agreed that they must intervene in cases of AUD and more than 80% considered that the prognosis for AUD depends on its proper management (25). In Denmark, the analysis of the perceptions and attitudes of university students showed that these students considered alcohol as an essential part of their lives and most of them agreed that increasing restrictions would be necessary (26). With regards to their attitudes, some of them suggested the need for secondary and tertiary prevention and intervention focused on persons at risk, while others suggested the need for primary universal prevention (27).

In relation to the stage of change which they are in when providing patients with assistance, according to Daley *et al*. (28), most of our respondents were in a stage of change at the time (58.2%), with a significantly higher percentage of medical students than dentistry students finding themselves in this situation. As far as we can see, no further studies have been conducted regarding this topic. Diana Ramos *et al*. studied the stages of change in relation to encouraging patients to give up the habit and recommended adapting the strategies to the stage of change which they students were in, suggesting using cognitive, affective and evaluative techniques in the early stages (pre-contemplation and contemplation) in order for them to progress through those stages (29). We consider reinforcing the syllabuses of these degrees to be fundamental since it has been observed that as the student’s knowledge of general pathology improves, the stage of change that the students finds themselves in also improves.

A lack of specific literature must be highlighted as one of the most significant limitations in this study. We believe that collaboration between groups could generate a new target for public health and a new path towards more publications. This has been achieved by other researchers who have carried out work on other risk factors in order to integrate this sensitive issues in day-to-day practice, such as Daley *et al*. with HPV (28) or Ramseier *et al*. with tobacco (30), in the case of AUD Miller *et al*. studies are highlighted (13). Likewise, we also believe that, due to the lack of literature on the matter, we should seize the opportunity to take action on what is certainly a matter of urgency.

## Conclusions

Most of the respondents presented with AUD, 75% of whom were women. The female gender is an independent risk factor for AUD (OR=2.63). Most of the participants’ knowledge of alcohol morbidity was considered to be insufficient. Globally, the participants’ level of knowledge was low, although this improved during the most advanced courses, with dentistry students presenting greater gaps in knowledge in terms of general pathology and medical students in terms of oral pathology. Practically all of the students agreed that identifying at-risk drinkers and providing them with assistance with this issue forms part of their work. With regards to their attitudes to AUD, the majority were in a stage of change (58.2%) and we observed that as their knowledge of general pathology improved, so too did the stage that they were in at that precise moment. The syllabuses must be reviewed in terms of their content on alcohol issues, and training programs must be implemented, which should be differentiated by gender in both degrees, and must emphasise morbidity related to alcohol and the knowledge about the tools and strategies for detecting AUD.
